# Age as a Prognostic Factor in Patients with Ewing Sarcoma—The Polish Sarcoma Group Experience

**DOI:** 10.3390/jcm10163627

**Published:** 2021-08-17

**Authors:** Paulina Jagodzińska-Mucha, Anna Raciborska, Hanna Koseła-Paterczyk, Katarzyna Kozak, Katarzyna Bilska, Tomasz Świtaj, Sławomir Falkowski, Anna Dawidowska, Piotr Rutkowski, Iwona Ługowska

**Affiliations:** 1 Department of Soft Tissue/Bone Sarcoma and Melanoma, Maria Sklodowska-Curie National Research Institute of Oncology, 02-781 Warsaw, Poland; hanna.kosela-paterczyk@pib-nio.pl (H.K.-P.); katarzyna.kozak@pib-nio.pl (K.K.); tomasz.switaj@pib-nio.pl (T.Ś.); slawomir.falkowski@pib-nio.pl (S.F.); piotr.rutkowski@pib-nio.pl (P.R.); iwona.lugowska@pib-nio.pl (I.Ł.); 2 Department of Oncology and Surgical Oncology for Children and Youth, Mother and Child Institute, 01-211 Warsaw, Poland; anna.raciborska@hoga.pl (A.R.); katarzyna.bilska@imid.med.pl (K.B.); 3 Early Phase Clinical Trials Unit, Maria Sklodowska-Curie National Research Institute of Oncology, 02-781 Warsaw, Poland; anna.dawidowska@pib-nio.pl

**Keywords:** Ewing sarcoma, prognostic factors, treatment outcomes

## Abstract

Ewing sarcoma (ES) is a rare and aggressive disease that requires multidisciplinary treatment with the use of chemotherapy, radiotherapy, and surgery. Our retrospective study aimed to analyze the prognostic factors and treatment results in different age groups of patients. Between 1998 and 2018, 569 patients with ES were treated in two referral centers. The patients were divided into four age groups (≤10 years; 11–18 years; 19–25, and >25). The treatment results and prognostic factors were assessed for each group. For statistical analyses, we used the Chi2 test, the Kaplan–Meier estimator with a log-rank test, and the multivariate Cox model. Five-year overall survival (OS) rate was 56%. In the age subgroups: ≤10 years, 11–18 years, 19–25 years, and >25 years, the 5-year OS rates were 75%, 58%, 41%, and 52%, respectively. Favorable prognostic factors: female gender (*p* = 0.024), non-axial localization (*p* = 0.005), VIDE regimen (*p* < 0.001), and surgery as a local treatment (*p* < 0.001) dominated in the group ≤10 years. In multivariate analysis, male (HR = 1.53), axial localization (HR = 1.46), M1 status at presentation (HR = 2.64), and age > 10 years (HR = 2.29) were associated with shorter OS. The treatment results in ES are significantly better in children aged ≤10 years; the challenge is to provide therapy for adolescents and young adults. The diagnostics and treatment of ES patients must be provided in referral centers.

## 1. Introduction

Ewing sarcoma (ES) is the third most common primary malignant bone tumor that most often affects children and patients in the second decade of life [1,2]. Skeletal localization predominates especially in young populations (80%), while localization in the soft tissue is more common in older patients (>75%) [3,4]. A more frequent occurrence is also observed in men and the Caucasian race [5,6]. The most common localizations of the tumor are the pelvic area and long bones of extremities with a predominance of the central femur [7,8]. About a quarter of patients have metastatic disease at the time of diagnosis [9]. Metastatic disease is most often found in the lungs (50%), bones (25%), and bone marrow (20%) [10,11].

Multiple prognostic factors in ES have been reported, such as age, gender, localization, volume and size of the primary tumor, presence of metastasis, treatment regimens, and a baseline level of hemoglobin or lactate dehydrogenase [8,11,12].

The chemotherapy (CHT) in ES was introduced in the 1960s; since then, the prognosis of patients with the localized disease has significantly improved [13,14,15]. Five-year overall survival (OS) rates increased from 10% for patients who received radiotherapy (RTH) alone to 55–65% for patients who received combined therapy with surgery, multidrug CHT, and RTH [6,16]. Despite multimodal treatment, patients with the disseminated disease still have a grim prognosis, and 5-year OS rates are only about 20% [11,12].

The most challenging population, considering diagnosis and treatment, is the adolescents and young adults group (AYA) because those patients may be treated in different oncological sites (for children or adults), and further follow-up is difficult, also for logistical reasons. The published data have shown that outcomes for older patients remain poor [17,18]. It is worth noting that toxicity of systemic therapy in adult patients is more frequent, resulting in reduced chemotherapy doses and a correlation with inferior outcomes [19,20].

This retrospective study aimed to analyze treatment results and prognostic factors of ES depending on the age.

## 2. Materials and Methods

### 2.1. Patients and Data Collections

We analyzed the electronic medical records of patients diagnosed with ES between 1998 and 2018 from two reference centers: Maria Sklodowska-Curie National Research Institute of Oncology in Warsaw (MSCNRIO), where adult patients were treated, and the Mother and Child Institute, Warsaw, where the pediatric population was treated. AYA were treated in both institutions. In all cases, histopathological examination was provided by the sarcoma expert pathologist from both institutions. The pathological confirmation of diagnosis (second opinion) was required before commencing treatment for all patients diagnosed outside the hospitals mentioned above. When possible, the diagnosis was confirmed by cytogenetic evaluation of *EWSR* fusion; however, most patients treated in the early 2000s did not have such examinations. We have tried to perform a re-analysis of *EWSR-1* by fluorescence in situ hybridization (FISH) as standard techniques at the time of this study, but due to the low quality of old FFPE samples, we were not able to obtain reliable results. For adult patients, approximately 50% had confirmed *EWSR* translocation, but due to technical restrictions only in recent years, fusion partners for *EWSR* gene were evaluated.

All consecutive patients, with good general condition (ECOG Performance Status < 2) treated in one of our institutions, regardless of the disease stage, were included in the analysis.

Clinicopathologic features including age, gender, localization and site of the primary tumor, metastases at diagnosis, localization of metastases, the presence of a pathological fracture, and treatment modality were collected. All patients were divided into four age subgroups: two subgroups of the pediatric population (≤10 years old, 11–18 years old) and two subgroups of adults (19–25 years old and >25 years old), which were analyzed in the context of prognostic factors based on collected clinicopathological features. Such division was justified by the differences in treatment modalities between the institutions from which the clinical data were collected. The differences in the treatment approach depending on the group ages were reported; in most patients < 18 years old, the treatment was based on EURO EWING protocols, and the patients ≥ 18 years old received different regimens of multidrug CHT based on doxorubicin, vincristine, cyclophosphamide, ifosfamide, etoposide, and dactinomycin according to existing recommendations The details of treatment regimens are described below.

The combined therapy for patients < 18 years was as follows: neoadjuvant CHT with six cycles of vincristine, ifosfamide, doxorubicin, and etoposide (VIDE), followed by surgical resection. Radiation therapy at a 45–54-Gy dose was applied in case of inoperable tumors or after non-radical surgery or when the histological response to neoadjuvant CHT was insufficient (<90% necrosis found in the postoperative specimen). After local treatment, adjuvant CHT was administered based on vincristine, dactinomycin, and cyclophosphamide or ifosfamide (VAI/VAC). Patients with a poor prognosis, due to metastatic disease or inadequate histological response to preoperative treatment, additionally received high-dose CHT followed by transplantation of hematopoietic stem cells. Chemotherapy did not need to be discontinued due to drug-related toxicity. There were also no toxic deaths.

The therapy for patients ≥18 years was: after 3–6 cycles of induction CHT (VIDE, EI/AC, or other schemes of multidrug protocol with doxorubicin, vincristine, cyclophosphamide, ifosfamide, etoposide, and dactinomycin) patients underwent local treatment with surgery ± RTH. As for perioperative, 55–60-Gy of RTH in conventional fractions of 2 Gy once per day was performed in most adult patients based on the risk factors. For patients treated with palliative intent, only chemotherapy based on the drugs mentioned above was administered.

### 2.2. Statistical Analyses

Statistical calculations were performed with the software package SPSS v. 19.0 (PL). Patient demographics, tumor characteristics, and treatment details were analyzed descriptively. The primary objective of the study was to assess the possible relationships between treatment results and clinic-pathological factors in age groups; therefore, the Chi2 tests were applied. The overall survival (OS) was estimated according to the Kaplan–Meier method, and log-rank tests were used for comparison. The OS time was calculated from diagnosis to the most recent follow-up or death. The death dates were confirmed in the Polish Death Registry using National Cancer Registry. In the next step, we performed the multivariate analysis using the Cox proportional hazards regression model. The Cox model included variables statistically significant at the *p*-value level of 0.1 or less in the univariate analysis. The differences were considered statistically significant if the *p*-value was < 0.05.

## 3. Results

### 3.1. Patients Characteristics and Treatment Outcomes

We retrospectively analyzed the medical records of 569 patients with ES diagnosed and treated between 1998–2018 in two reference sarcoma centers involved in the Polish Sarcoma Group: 211 patients from MSCNRIO and 358 patients from the Mother and Child Institute, Warsaw.

The primary tumor location was axial in 322 cases (57%); the remaining 247 patients (43%) had tumors localized in the extremities. Thirty-one patients with extremities localization (13%) had a pathological fracture before local treatment. A total of 356 patients (63%) had the disease locally advanced at diagnosis, while in 213 cases (37%), metastatic disease at baseline was diagnosed. Lung metastases were diagnosed in 97 patients (46%). In the remaining 116 cases (54%), metastases were presented in other locations, including the skeleton, the bone marrow, and less frequent soft tissue, mediastinum, central nervous system, and peritoneum. The patient characteristics are shown in Table 1.

In the analyzed group, 560 patients (98%) received chemotherapy. In 342 cases (60%), VIDE regimen was used for treatment; in 218 cases (38%), other chemotherapy schemes were applied. Table 2 lists chemotherapy regimens used for treatment in particular age subgroups. High-dose CHT with the following transplantation of hematopoietic stem cells was used in 87 patients <25 years old (15%). Five hundred and thirteen patients (90%) were qualified for local treatment (radiotherapy ± surgery). The rest of the 56 patients (10%) were disqualified from local treatment and received only chemotherapy with palliative intend. Details of chemotherapy types are presented in Table 2.

The five-year OS rate in the analyzed group of 569 patients was 55.7%. At the time of analysis, 253 patients (44%) died, and 246 patients (43%) had disease progression on/after the first line of treatment. The median follow-up was 42 months (range: 2–259 months).

### 3.2. The Univariate Analysis

The following factors had the negative impact on OS: male gender (*p* = 0.006), age > 10 years old (*p* < 0.001), axial tumor localization (*p* < 0.001), presence of metastases at the time of diagnosis (*p* < 0.001), extrapulmonary localization of metastases at the time of diagnosis (*p* < 0.0001), other than VIDE chemotherapy regimen used for first-line treatment (*p* < 0.001), no local treatment (*p* < 0.001), and inoperable disease (*p* < 0.001). Five-year OS rates for specific prognostic factors are shown in Table 3.

### 3.3. The Multivariate Analysis

**Figure 1:**
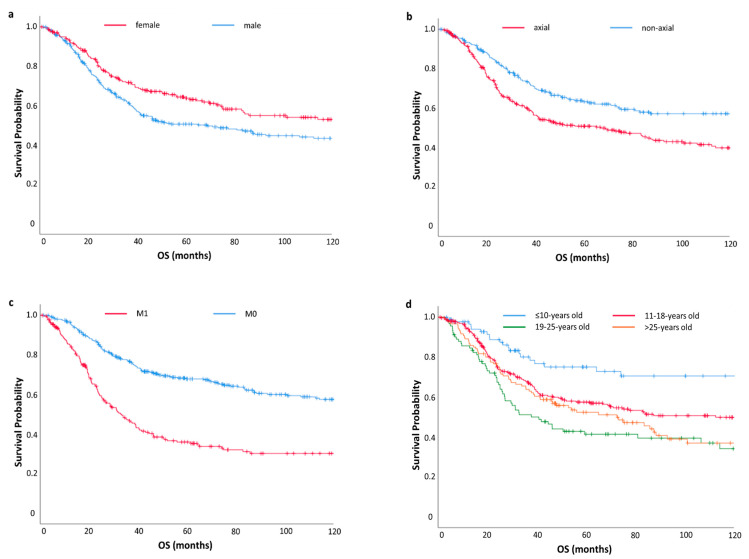
Overall survival (OS) in the study population stratified by gender (**a**), primary tumor location (**b**), metastatic status (**c**), and age (**d**).

In multivariate analysis, the male gender (HR = 1.53; 95% CI: 1.17–1.99, *p* = 0.002), axial localization of primary (HR = 1.46; 95% CI: 1.12–1.90, *p* = 0.005), and presence of metastases status at presentation (HR = 2.64; 95%CI 2.05–3.41, *p* < 0.001) were associated with shorter OS. Patients aged >10 y (three age subgroups: 11–18 years old, 19–25 years old and >25 years old) had poorer prognosis than patients ≤10 years old (HR = 2.27; 95% CI 1.41–3.72, *p* = 0.001) (Table 4) (Figure 1).

### 3.4. Age Subgroups Analyses

In the analyzed age subgroups (≤10 years old, 11–18 years old, 19–25 years old, >25 years old), differences in the incidence of specific prognostic factors were observed. It should be noted that prognostic factors associated with a good prognosis predominated in patients ≤10 years old. In the other three age subgroups (11–18 years old, 19–25 years old, and >25 years old), negative prognostic factors were observed more frequently. Detailed characteristics of each age group are presented in Table 5.

In the group of patients ≤10 years old, prognostic factors related to longer OS dominated. The majority of patients in this group were females (57%) and had a non-axial localization of the primary tumor (55%). Intensive systemic chemotherapy based on VIDE regimen (91%) combined with auto-BMT (autologous bone marrow transplantation) (24%), and surgery (81%) dominated in the group of the youngest patients. It should also be noted that 78% of patients from this group were still alive at the time of analysis.

In the group of patients >10 years old, unfavorable prognostic factors occurred more frequently. This group of patients was dominated by males (60%) and axial localization of the primary (59%). Moreover, radiotherapy was given more often as a single treatment modality in inoperable cases (23% vs. 7%), as well as disqualification from surgical treatment was more common (33% vs. 13%), which was associated with a higher tumor burden at diagnosis in older patients. It is worth emphasizing that auto-BMT was not applied to any patients >25 years old.

On the other hand, our analysis includes only 20% of patients with disseminated disease >25 years old, while in the group of patients ≤25 years old, there are more patients with metastases (40%). Patients aged >25 years old had more frequently bad prognostic factors such as worst performance status (WHO ≥ 2) and high tumor burden at diagnosis. This group of patients was under oncological care outside the reference centers and was excluded from analysis due to no access to their electronic medical records.

Disease progression during or after first-line treatment occurred more often in patients >10 years old (45% vs. 32%). At the time of the analysis, the highest number of deaths occurred in patients >10 years (50% vs. 22%).

## 4. Discussion

This retrospective analysis aimed to assess treatment results and prognostic factors in different age groups of patients diagnosed with ES based on the data from two reference centers in Poland. We have found that age ≤ 10 years, female, non-axial location of the primary, and an absence of metastases at diagnosis had an independent favorable impact on the survival. In our cohort, patients ≤10 years old demonstrated a higher proportion of favorable prognostic factors when compared to the other subgroups. The results of our analysis based on 569 patients are similar to those few studies that have already been published and showed that age has a significant and independent impact on patients’ prognosis.

In the previously published retrospective analyses, the outcomes of treatment among adolescents and young adults (AYA) were worse than in children [17,21,22]. Moreover, a higher proportion of unfavorable prognostic factors were observed in older patients [17,21,22]. Although the National Cancer Institute (NCI) guidelines define adults and young adults (AYA) between 15 and 39 years old, the age categories vary between studies what may determine the results.

Cotterill et al., in their study based on 975 patients, observed that negative prognostic factors, such as male, primary location in the pelvis, and high tumor burden, were predominant in the group of patients > 15 years old [8].

Another retrospective analysis based on 2930 patients from a German central database confirmed that high tumor burden, axial localization, and disseminated disease occurred more frequently in the population of patients > 10 years old [17]. It has to be underlined that the diagnosis of patients with an axial localization was often delayed due to the late occurrence of symptoms. It also resulted in a higher tumor burden at the time of diagnosis and later detection of metastases.

Our analysis included four age groups. The pediatric population was divided into two subgroups: ≤10 years old and 11–18 years old. The adult population was divided into two subgroups: 19–25 years old and >25 years old. This division was determined by differences in collected data between the pediatric and adult populations treated in two independent institutions.

Based on the available literature, it is known that AYA has a poorer prognosis than the pediatric population, although the treatment is based on similar regimens [17,22,23]. CHT used in ES includes the combination of vincristine, dactinomycin, doxorubicin, etoposide, cyclophosphamide, and ifosfamide [24]. Based on the recommendations of the National Comprehensive Cancer Network (NCCN) in localized ES, the most commonly used regimens are vincristine, cyclophosphamide, and doxorubicin (VCD), alternating with ifosfamide and etoposide (IE); vincristine, doxorubicin, and ifosfamide (VAI), more commonly used in North America; or vincristine, ifosfamide, doxorubicin, and etoposide (VIDE), which are popular in Europe [24,25]. Our analysis showed some differences between the treatment strategies of young and adult patients. According to Euro-E.W.I.N.G.99 and EWING-2008, administration of busulphan/melphalan in a high-risk group of patients improves treatment outcomes [26,27]. In our analysis, this kind of therapy was used only in patients under 18 years old, which could impact the improvement of treatment results. Such therapy in adult patients is not a standard of care due to insufficient data in the context of toxicity and efficacy. Moreover, a higher toxicity of systemic treatment is observed in adult patients, resulting in longer intervals between cycles and, more often, reduction in CHT doses [19,28]. J. Zhang et al., as well as Womer et al., showed that dose density, the number of cycles, and frequency of CHT courses are independent prognostic factors [19,28].

Considering the different treatment regimens across institutions, further research is needed to standardize treatment guidelines worldwide. The first results of the EURO EWING 2012 trial comparing two CHT regimens (VCD/IE vs. VIDE) were presented at CTOS conference in Tokyo in 2019 and ASCO conference in Chicago in 2020 [29]. The study included 640 patients aged 5–50 years old with newly diagnosed or metastatic Ewing sarcoma. The influence on survival depending on various prognostic factors, including gender, age, status and localization of metastases, tumor volume, and country, were analyzed, but no effect on outcomes was found [29].

It has been proven that OS is better with VCD/IE regardless of age and other prognostic factors [29]. This is undoubtedly a significant step forward in the context of developing a new clinical trial to improve treatment results.

Developing a prognostic model is very challenging due to the rarity and heterogeneity of the Ewing Sarcoma and requires multicenter cooperation. Besides age, our multivariate analysis confirmed the modest survival advantage in females. However, considering the population data, the impact of gender on the prognosis is uncertain. Retrospective analysis based on the Surveillance Epidemiology and End Results (SEER) database showed statistically significant better results of 5-year OS rates for women [6,30]. However, there were no differences in survival between both genders in other publications [31].

It is well known that metastases at diagnosis are an independent and negative prognostic factor [25,27]. The long-term OS for patients with the disseminated disease is still only 20%; however, patients with isolated lung metastases have a better outcome than patients with extrapulmonary disease [25,27]. In our analysis, metastases at baseline were more common in patients aged 10–18 years old than in patients above 25 years old (45% vs. 20%, respectively). It could be biased because about 20% of older patients with stage IV and ECOG performance status >1, assigned only to palliative treatment, were excluded from this analysis due to treatment outside the reference centers and limited data about their follow-up.

Other significant predictors of ES are the localization of the primary tumor and its volume [22,32]. Our analysis indicates that non-axial localization of the primary was more common in patients ≤10 years old. Localization in the extremities allows for an earlier diagnosis and treatment. Patients with axial localization had the poorest prognosis due to higher tumor volume and more advanced disease at baseline. Due to the lack of sufficient data concerning the primary tumor size in a high percentage of patients, we could not include this factor in our analysis.

It has to be noted that some studies reported pathologic response to neoadjuvant chemotherapy as an important prognostic factor [33]. We have not included this parameter because of the lack of data for a significant proportion of patients. Patients included in the analysis were treated for a long time (between 1998–2018). For many of them, pathologic response was not reported in the histopathological reports in a consistent, standardized manner. No systematic guidelines for reporting the pathologic responses were available in the 1990s and early 2000s, thus it has not been comprehensively assessed. Moreover, treatment regimens and doses of chemotherapy differ significantly between pediatric and adult populations, affecting the degree of pathological responses. To reduce bias associated with this issue and a significant number of cases with missing data about responses, we decided not to include this parameter.

Besides clinicopathological factors presented in our analysis that impact patients’ survival, there are mutational or signaling pathway differences between age subgroups associated with prognosis [34]. The number of genomic alterations and the appearance of new mutations increases with age, which could explain the more aggressive nature of the disease in the AYA population [34]. Conventional Ewing sarcomas are characterized by the EWSR1-ETS translocation [34,35]. Ewing sarcomas with EWSR1-non-ETS translocation, known as Ewing-like sarcomas, are significantly different clinically and pathologically. Therefore, the WHO classification of 2020 distinguished it as a separate disease entity [36,37]. Tsuda et al., in their retrospective analyses, showed that EWSR1-FEV and EWSR1-NFATC2 fusions were associated with older age, compared to the EWSR1-FLI translocation that predominated in a young population [37]. Primary axial and pelvic locations were more common in patients with EWSR1-FLI1, while extremity localization of primary was more common in patients with EWSR1-NFATC2. Three-year OS was significantly better in EWSR1-FLI patients (91%) than EWSR1-nonETS patients (60%), and the higher proportion of metastases dominated in the group of patients with EWSR1-nonETS fusions [37]. However, the influence of biological and molecular factors is not sufficiently described and requires further investigation [34,35,36,37].

Regarding the retrospective nature of this study, it has several limitations. Firstly, the lower proportion of patients with disseminated disease in patients >25 years old is noticeable. Approximately 20% of adults were qualified for palliative treatment outside the reference center and were excluded from the analysis due to incomplete data. Moreover, treatment was based on different chemotherapy regimens, and the indications for radiotherapy were different in pediatrics and adults. Given the lack of complete data, tumor size, presence, and type of EWSR translocation, chemotherapy doses, density, and toxicity were not explored in the context of survival.

## 5. Conclusions

To summarize, we reported a large cohort of pediatric and adult patients with ES treated in two reference sarcoma centers in Poland.

Our study showed that several prognostic factors, such as age, gender, metastases status at baseline, tumor location, and treatment modality, influenced survival. The prognostic factors related to longer OS dominated in the group of patients ≤10 years old. Biological differences between age subgroups should be investigated in the future to explain the impact on the survival of these groups of patients with ES [38,39,40]. Our analysis can help develop a predictive model for patients with ES, which would allow adjusting the treatment depending on the risk of recurrence or disease progression in different age groups.

## Figures and Tables

**Table 1:** Patients’ clinicopathological characteristics.

Clinicopathological Factors		Number of Patients N (%)
Sex	female	243 (43%)
	male	326 (57%)
Age at diagnosis	≤10 years	91 (16%)
	11–18 years	267 (47%)
	19–25 years	91 (16%)
	>25 years	120 (21%)
Tumor localization	axial	322 (57%)
	non-axial	247 (43%)
Metastases at diagnosis	M0	356 (63%)
	M1	213 (37%)
Localization of metastases	M0	356 (63%)
	Lung	97 (17%)
	other	116 (20%)
Pathological fracture	Yes	31 (5%)
	No	533 (94%)
	missing data	5 (1%)
Clinicopathological factors		Number of patients N (%)
Chemotherapy regimen	no CHT	9 (2%)
	other	218 (38%)
	VIDE	342 (60%)
Local treatment	Surgery	184 (32%)
	RTH+surgery	213 (38%)
	RTH	116 (20%)
	No	56 (10%)
Type of surgery	LSS	375 (66%)
	amputation	33 (6%)
	No	161 (28%)
Auto-BMT	Yes	87 (15%)
	No	482 (85%)
Disease progression	Yes	246 (43%)
	No	323 (57%)
Survival status	Deceased	253 (44%)
	Alive	316 (56%)

CHT—chemotherapy; RTH—radiotherapy; VIDE—Vincristine, Ifosfamide, Doxorubicin, Etoposide; LSS—limb-sparing surgery, auto-BMT—autologous bone marrow transplantation.

**Table 2:** Chemotherapy regimens used in the study population.

Chemotherapy Regimen	Age Subgroups, Number of Patients 560 (9 Patients did not Received CHT)			
	≤10 Years Old (88 Patients)	11–18 Years Old (264 Patients)	19–25 Years Old (90 Patients)	>25 Years Old (118 Patients)
EI/AC	0 (0%)	1 (<1%)	34 (38%)	50 (42%)
VADRIAC/EIAO	1 (1%)	15 (6%)	33 (37%)	42 (36%)
VIDE	83 (95%)	234 (89%)	15 (17%)	10 (8%)
EVAIA	1 (1%)	9 (3%)	5 (5%)	7 (6%)
EI	0 (0%)	0 (0%)	3 (3%)	7 (6%)
other	3 (3%)	5 (2%)	0 (0%)	2 (2%)

All cycles given every 21 days: EI/AC: 3 cycles of Ifosfamide, 2 g/m^2^ on days 1–3; Etoposide 150 mg/m^2^ on days 1–3; Cyclophosphamide 1.5 g/m^2^ on day 5; Doxorubicin 45 mg/m^2^ on day 5; local control measures: weekly Vincristine 1.5 mg/m^2^; biweekly Dactinomycin 0.5 mg/m^2^; maintenance therapy—4 cycles each of of Ifosfamide, 2 g/m^2^ on days 1–5; Etoposide 150 mg/m^2^ on days 1–5 alternating with Cyclophosphamide 1.5 g/m^2^; Doxorubicin 60 mg/m^2^ (infusion for 24 h) VADRIAC/EIAO: Vincristine 1.5 mg/m^2^ on day 1 (max 2 mg); Doxorubicin 37.5 mg/m^2^ on days 1–2; Cyclophosphamide 600 mg/m^2^ on days 1–2 alternating with Etoposide 150 mg/m^2^ on days 1–4 Vincristine 1.5 mg/m^2^ on day 1 Dactinomycin 0.5 mg/m^2^ on days 1–2; Ifosfamide, 1.7 g/m^2^ on days 1–4 VIDE: Vincristine 1.5 mg/m^2^ on day 1 (max 2 mg); Ifosfamide, 3 g/m^2^ on days 1–3; Doxorubicin 20 mg/m^2^ on days 1–3; Etoposide 150 mg/m^2^ on days 1–3 EVAIA: Etoposide 150 mg/m^2^ on days 1–3; Vincristine 1.5 mg/m^2^ on day 1; Doxorubicin 30 mg/m^2^ alternating with Dactinomycin 0.5 mg/m^2^ on days 1–3; Ifosfamide, 2 g/m^2^ on days 1–3 EI: Etoposide 100 mg/m^2^ on days 1–5; Ifosfamide, 1.8 g/m^2^ on days 1–5.

**Table 3:** Five-year OS for specific prognostic factors in a group of 569 patients.

Prognostic Factor		5-Year OS Rate	*p*-Value
Sex	Female	64%	0.006
	Male	51%	
Age	≤10 years old	75%	<0.001
	11–18 years old	58%	
	19–25 years old	41%	
	>25 years old	52%	
Tumour location	Axial	51%	<0.001
	non-axial	63%	
Metastases	M0	68%	<0.001
	M1	36%	
Localization of metastases	M0	68%	<0.001
	Lung	48%	
	Other	24%	
Pathological fracture	Yes	49%	0.569
	No	56%	
Chemotherapy regimen	no CHT	44%	<0.001
	Other	46%	
	VIDE	64%	
Local treatment	Surgery	69%	<0.001
	RTH+Surgery	63%	
	RTH	41%	
	no	19%	
Type of surgery	LSS	68%	<0.001
	Amputation	46%	
	No	32%	
Auto-BMT	Yes	59%	0.198
	No	56%	

CHT—chemotherapy; RTH—radiotherapy; VIDE—Vincristine, Ifosfamide, Doxorubicin, Etoposide; LSS—limb-sparing surgery, auto-BMT—autologous bone marrow transplantation

**Table 4:** Cox proportional hazard model for survival.

Variables in the Equation	*p*	Exp (B)	95% CI for Exp (B)	
			Lower	Upper
Sex: male (vs. female)	0.002	1.525	1.170	1.987
Tumour location: axial (vs. non-axial)	0.005	1.458	1.120	1.898
Metastases: M1 (vs. M0)	<0.001	2.641	2.048	3.407
Pathological fracture	0.290	1.330	0.784	2.259
Age: >10y (11–18 years old, 19–25 years old and >25 years old) vs. ≤10y	0.001	2.286	1.407	3.715

**Table 5:** Prognostic factors depending on the age subgroups.

Prognostic Factors		Age Subgroups				
		<10 Years	10–18 Years	18–25 Years	>25 Years	*p*
Sex	Female	52 (57%)	109 (41%)	35 (38%)	47 (39%)	0.024
	Male	39 (43%)	158 (59%)	56 (62%)	73 (61%)	
Tumour location	Axial	41 (45%)	143 (54%)	61 (67%)	77 (64%)	0.005
	non-axial	50 (55%)	124 (46%)	30 (33%)	43 (36%)	
Metastases	M0	56 (62%)	148 (55%)	56 (61%)	96 (80%)	<0.001
	M1	35 (38%)	119 (45%)	35 (39%)	24 (20%)	
Localization of metastases	M0	56 (62%)	148 (55%)	56 (61%)	96 (80%)	<0.001
	Lung	20 (22%)	50 (19%)	16 (18%)	11 (9%)	
	Other	15 (16%)	69 (26%)	19 (21%)	13 (11%)	
Pathological fracture	Yes	5 (6%)	17 (6%)	2 (2%)	7 (6%)	0.524
	No	85 (94%)	250 (94%)	87 (98%)	111 (94%)	
Chemotherapy regimen	no CHT	3 (3%)	3 (1%)	1 (1%)	2 (2%)	<0.001
	Other	5 (6%)	30 (11%)	75 (82%)	108 (90%)	
	VIDE	83 (91%)	234 (88%)	15 (17%)	10 (8%)	
Local treatment	Surgery	54 (59%)	110 (41%)	10 (11%)	10 (8%)	<0.001
	RTH + surgery	25 (27%)	91 (34%)	41 (45%)	56 (47%)	
	RTH	6 (7%)	42 (16%)	24 (26%)	44 (37%)	
	No	6 (7%)	24 (9%)	16 (18%)	10 (8%)	
Type of surgery	LSS	79 (87%)	191 (71%)	48 (53%)	57 (48%)	<0.001
	Amputation	5 (2%)	18 (7%)	3 (3%)	10 (8%)	
	No	10 (11%)	58 (22%)	40 (44%)	53 (44%)	
Auto-BMT	Yes	22 (24%)	60 (22%)	5 (5%)	0 (0%)	<0.001
	No	69 (76%)	207 (78%)	86 (95%)	120 (100%)	
Disease progression	Yes	29 (32%)	106 (40%)	50 (55%)	61 (51%)	0.003
	No	62 (68%)	161 (60%	41 (45%)	59 (49%)	
Died	Yes	20 (22%)	113 (42%)	54 (59%)	66 (55%)	<0.001
	No	71 (78%)	154 (58%)	37 (41%)	45 (45%)	

CHT—chemotherapy; RTH—radiotherapy; VIDE—Vincristine, Ifosfamide, Doxorubicin, Etoposide; LSS—limb-sparing surgery, auto-BMT—autologous bone marrow transplantation

## Data Availability

Data will be available from the corresponding author upon reasonable request.

## References

[1] Casali P.G., Bielack S., Guidelines C.P. (2018). Bone sarcomas: ESMO–PaedCan–EURACAN Clinical Practice Guidelines for diagnosis, treatment and Clinical Practice Guidelines. Ann. Oncol..

[2] Tsokos M., Alaggio R.D. (2012). Ewing sarcoma/peripheral primitive neuroectodermal tumor and related tumors. Pediatr. Dev. Pathol..

[3] Cash T., McIlvaine E. (2016). Comparison of Clinical Features and Outcomes in Patients with Extraskeletal Versus Skeletal Localized Ewing Sarcoma: A Report from the Children’s Oncology Group. Pediatr. Blood Cancer.

[4] Lee J.A., Kim D.H. (2010). Soft-tissue Ewing sarcoma in a low-incidence population: Comparison to skeletal Ewing sarcoma for clinical characteristics and treatment outcome. Jpn. J. Clin. Oncol..

[5] Damron T.A., Ward W.G. (2007). Osteosarcoma, chondrosarcoma, and Ewing’s sarcoma: National cancer data base report. Clin. Orthop. Relat. Res..

[6] Esiashvili N., Goodman M. (2008). Changes in Incidence and Survival of Ewing Sarcoma Patients Over the Past 3 Decades. J. Pediatr. Hematol. Oncol..

[7] Bosma S.E., Ayu O. (2018). Prognostic factors for survival in Ewing sarcoma: A systematic review. Surg. Oncol..

[8] Cotterill S.J., Ahrens S. (2000). Prognostic factors in Ewing’s tumor of bone: Analysis of 975 patients from the european intergroup cooperative Ewing’s sarcoma study group. J. Clin. Oncol..

[9] Bernstein M., Kovar H. (2006). Ewing’ s Sarcoma Family of Tumors: Current management. Oncologist.

[10] Tan Q.T., Teo J.Y. (2016). A case of small bowel metastasis from spinal Ewing sarcoma causing intussusception in an adult female. World J. Surg. Oncol..

[11] Balamuth N.J., Womer R.B. (2010). Ewing’ s sarcoma. Lancet Oncol..

[12] Duchman K.R., Gao Y. (2015). Prognostic factors for survival in patients with Ewing’s sarcoma using the surveillance, epidemiology, and end results (SEER) program database. Cancer Epidemiol..

[13] Pinkel D. (1961). Cyclophosphamide in children with cancer. Cancer.

[14] Sutow W.W., Sullivan M.P. (1962). Cyclophosphamide therapy in children with Ewing’s sarcoma. Cancer Chemother. Rep..

[15] Jain S., Kapoor G. (2010). Chemotherapy in Ewing’s sarcoma. Indian J. Orthop..

[16] Rosen G., Wollner N. (1974). Disease-free survival in children with Ewing’s sarcoma treated with radiation therapy and adjuvant four-drug sequential chemotherapy. Cancer.

[17] Worch J., Ranft A. (2018). Age dependency of primary tumor sites and metastases in patients with Ewing sarcoma. Pediatr. Blood Cancer.

[18] Liu H., Wang J. (2018). Clinical Features and Prognostic Factors in Elderly Ewing Sarcoma Patients. Med. Sci. Monit..

[19] Zhang J., Huang Y. (2019). Impact of chemotherapy cycles and intervals on outcomes of nonspinal Ewing sarcoma in adults: A real-world experience. BMC Cancer.

[20] Gupta A.A., Pappo A. (2010). Clinical Outcome of Children and Adults With Localized Ewing Sarcoma. Cancer.

[21] Perisa M.P., Stanek J. (2020). Evaluating Age-related Disparity of Outcomes in Ewing Sarcoma Patients Treated at a Pediatric Academic Medical Center. J. Pediatr. Hematol. Oncol..

[22] Marina N., Granowetter L. (2015). Age, Tumor Characteristics, and Treatment Regimen as Event Predictors in Ewing: A Children’s Oncology Group Report. Sarcoma.

[23] Rotz S.J., Nagarajan R. (2017). Challenges in the Treatment of Sarcomas of Adolescents and Young Adults. J. Adolesc. Young Adult Oncol..

[24] (2016). National Comprehensive Cancer Network: Bone Cancer. http://www.nccn.org/professionals/physician_gls/pdf/bone.pdf.

[25] Wagner M.J., Livingston J.A. (2016). Chemotherapy for Bone Sarcoma in Adults. J. Oncol. Pract..

[26] Whelan J., Le Deley M.C. (2018). High-Dose Chemotherapy and Blood Autologous Stem-Cell Rescue Compared With Standard Chemotherapy in Localized High-Risk Ewing Sarcoma: Results of Euro-E.W.I.N.G.99 and Ewing-2008. J. Clin. Oncol..

[27] Umeda K., Miyamura T. (2021). Prognostic and therapeutic factors influencing the clinical outcome of metastatic Ewing sarcoma family of tumors: A retrospective report from the Japan Ewing Sarcoma Study Group. Pediatr. Blood Cancer.

[28] Womer R.B., West D.C. (2012). Randomized Controlled Trial of Interval-Compressed Chemotherapy for the Treatment of Localized Ewing Sarcoma: A Report From the Children’ s Oncology Group. J. Clin. Oncol..

[29] Brennan B., Laura K.L. (2020). Comparison of two chemotherapy regimens in Ewing sarcoma (ES): Overall and subgroup results of the Euro Ewing 2012 randomized trial (EE2012). J. Clin. Oncol..

[30] Ries L.A.G., Smith M.A. (1999). Cancer Incidence and Survival among Children and Adolescents: United States SEER Program 1975–1995.

[31] Stiller C.A., Passmore S.J. (2006). Patterns of care and survival for patients aged under 40 years with bone sarcoma in Britain, 1980–1994. BMJ.

[32] Stachelek G.C., John A. (2021). Predictors of Recurrence and Patterns of Initial Failure in Localized Ewing Sarcoma: A Contemporary 20-Year Experience. Sarcoma.

[33] Albergo J.I., Gaston C.L. (2016). Ewing’s sarcoma: Only patients with 100% of necrosis after chemotherapy should be classified as having a good response. Bone Joint J..

[34] Hesla A.C., Papakonstantinou A. (2021). Current Status of Management and Outcome for Patients with Ewing Sarcoma. Cancers.

[35] Stefan K., Zöllner J.F. (2021). Ewing Sarcoma—Diagnosis, Treatment, Clinical Challenges and Future Perspectives. J. Clin. Med..

[36] Sbaraglia M., Righi A. (2020). Ewing sarcoma and Ewing-like tumors. Virchows Arch..

[37] Tsuda Y., Zhang L. (2020). The clinical heterogeneity of round cell sarcomas with EWSR1/FUS gene fusions: Impact of gene fusion type on clinical features and outcome. Genes Chromosom. Cancer.

[38] Verma V., Denniston K.A. (2017). A Comparison of Pediatric vs. Adult Patients with the Ewing Sarcoma Family of Tumors. Front. Oncol..

[39] Ahn H.K., Uhm J.E. (2011). Analysis of prognostic factors of pediatric-type sarcomas in adult patients. Oncology.

[40] Krakorova D.A., Kubackova K. (2017). Advantages in Prognosis of Adult Patients with Ewing Sarcoma: 11-years Experiences and Current Treatment Management. Pathol. Oncol Res..

